# A Streamlined Approach by a Combination of Bioindication and Geostatistical Methods for Assessing Air Contaminants and Their Effects on Human Health in Industrialized Areas: A Case Study in Southern Brazil

**DOI:** 10.3389/fpls.2017.01575

**Published:** 2017-09-20

**Authors:** Angélica B. Ferreira, Andreza P. Ribeiro, Maurício L. Ferreira, Cláudia T. Kniess, Cristiano C. Quaresma, Raffaele Lafortezza, José O. Santos, Mitiko Saiki, Paulo H. Saldiva

**Affiliations:** ^1^Institute of Technology and Research of Sergipe Aracaju, Brazil; ^2^Smart and Sustainable Cities, Nove de Julho University São Paulo, Brazil; ^3^Professional Masters in Environmental Management and Sustainability, Nove de Julho University São Paulo, Brazil; ^4^Agricultural and Environmental Sciences, University of Bari Bari, Italy; ^5^Center for Global Change and Earth Observations, Michigan State University, East Lansing MI, United States; ^6^Federal Institute of Sergipe Lagarto, Brazil; ^7^Center of the Nuclear Research Reactor, Nuclear and Energy Research Institute (IPEN) São Paulo, Brazil; ^8^Faculty of Medicine, University of São Paulo São Paulo, Brazil

**Keywords:** air pollution, environmental monitoring, geostatistical approach, industrial pollutants, urban impact

## Abstract

Industrialization in developing countries associated with urban growth results in a number of economic benefits, especially in small or medium-sized cities, but leads to a number of environmental and public health consequences. This problem is further aggravated when adequate infrastructure is lacking to monitor the environmental impacts left by industries and refineries. In this study, a new protocol was designed combining biomonitoring and geostatistics to evaluate the possible effects of shale industry emissions on human health and wellbeing. Futhermore, the traditional and expensive air quality method based on PM_2.5_ measuring was also used to validate the low-cost geostatistical approach. Chemical analysis was performed using Energy Dispersive X-ray Fluorescence Spectrometer (EDXRF) to measure inorganic elements in tree bark and shale retorted samples in São Mateus do Sul city, Southern Brazil. Fe, S, and Si were considered potential pollutants in the study area. Distribution maps of element concentrations were generated from the dataset and used to estimate the spatial behavior of Fe, S, and Si and the range from their hot spot(s), highlighting the regions sorrounding the shale refinery. This evidence was also demonstrated in the measurements of PM_2.5_ concentrations, which are in agreement with the information obtained from the biomonitoring and geostatistical model. Factor and descriptive analyses performed on the concentrations of tree bark contaminants suggest that Fe, S, and Si might be used as indicators of industrial emissions. The number of cases of respiratory diseases obtained from local basic health unit were used to assess a possible correlation between shale refinery emissions and cases of repiratory disease. These data are public and may be accessed on the website of the the Brazilian Ministry of Health. Significant associations were found between the health data and refinery activities. The combination of the spatial characterization of air pollution and clinical health data revealed that adverse effects were significant for individuals over 38 years of age. These results also suggest that a protocol designed to monitor urban air quality may be an effective and low-cost strategy in environmentally contaminated cities, especially in low- and middle-income countries.

## Introduction

A robust air quality management system is vital for protecting public health in the face of technological and climate change impacts. However, despite efforts from the United States Environmental Protection Agency (USEPA) to provide guidance on the use of sensors and data interpretation, in addition to promoting workshops with a focus on streamlining technologies, traditional networks with stationary facilities are still costly and complex (Snyder et al., 2013). Accordingly, the World Health Organization (World Health Organization [WHO], 2012) has encouraged the development of environmental studies to verify the feasibility of adopting simplified techniques such as bioindication/biomonitoring for measuring air pollution.

Both biological methods have been considered as low-cost and effective tools for identifying the impacts regarding external factors on ecosystems, by comparing unpolluted areas with polluted ones, or their consequences over the long term, in a specific location (Markert et al., 1997, 2003). Based on the receptor responses to environmental stress, some premises may be raised on the risks for human being (Mulgrew and Williams, 2009; Markert et al., 2011).

The difference between the methods lies in the fact that bioiondication approach supplys information on the quality of the environment, whereas quantitative aspects from environmental stresses, particularly due to chemical substances, are obtained by the biomonitoring approach (Markert et al., 1997, 2003).

Living organisms, such as plant leaves, lichens, moss and tree bark, are receptors of atmospheric contaminants (Markert et al., 2003; Schelle et al., 2008; Ferreira et al., 2012; Norouzi et al., 2015). With respect to tree barks, their suitability as bioindicator have been considered in critical areas for assessing air quality (Kuang et al., 2007; Schelle et al., 2008; Sawidis et al., 2011).

In this study, an atmospheric quality assessment of São Mateus do Sul City, Brazil, was performed adopting tree bark as a pollution bioindicator together with geographic and health datasets, since this city hosts a refinery that extracts shale from the soil for the production of oil, gas and sulfur by heating organic material.

During the cooling process observed in shale refineries, gas and vapors are emitted into the atmosphere releasing organic and inorganic chemical compounds that are harmful to the environment and human health. In all the stages of shale extraction (mining, transportation, and residue stockpiling), particulate matter is produced and carried by the wind reaching neighboring areas. Therefore, the type and level of pollutants determined in tree bark sample will enable to supply information on the air quality (Kuang et al., 2007; Sawidis et al., 2011) with reference to São Mateus do Sul.

The precise geographical coordinates of the sampling site allow verifying the spatial variation of the pollutants (Hoek et al., 2008). As a consequence of the economic benefits of the shale oil industry, São Mateus do Sul has geographically expanded toward the shale plant subjecting the surrounding population to industrial emissions. However, the area affected by the shale industry and the related health effects were not verified because air pollution measurements are not available in this area. Indeed, the potential sources of air pollution in areas without a qualified structure for measuring contaminants is quite a common phenomenon in developing countries (Norouzi et al., 2015; Moreira et al., 2016).

The overarching goal of our study is to devise a streamlined approach to assess air contaminants and their effects on human health in the industrialized area of São Mateus do Sul. We used the bioindincation method combined with a geostatistical model to trace elements of air pollution and identifying hot spots of contamination at large scale. Finally, we investigated the relationship between the spatial distribution of the hot spots and the health data related to respiratory disease in people living in the study area.

Our research features a novel approach – the “Attenuation of the Concentration Model” – (Wasserman and Queiroz, 2004; Ribeiro et al., 2013) that provides data similar to those obtained through conventional methods for monitoring air pollution based on measuring the composition of fine particulate matter (Brown et al., 2007).

## Materials and Methods

### Area of Study

**FIGURE 1:**
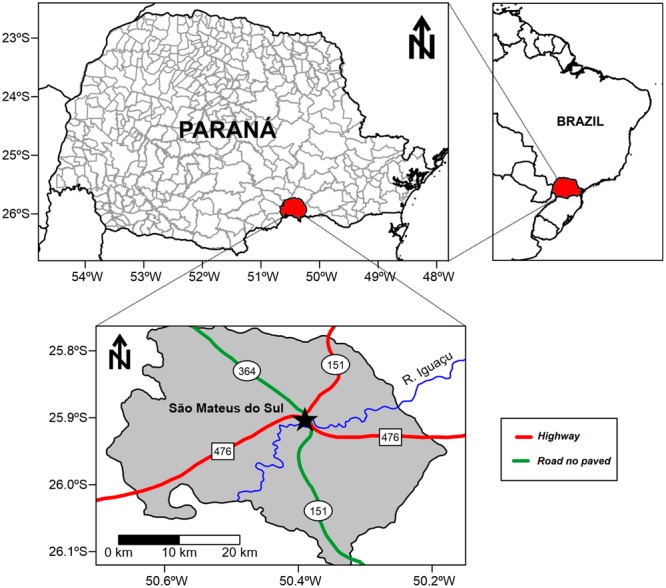
Location of the study area, São Mateus do Sul in the State of Paraná, Southern Brazil.

São Mateus do Sul is located in the southern region of Paraná State (latitude 25°44′S- 26°08′S, longitude 50°09′W-50°39′W) about 150 km from Curitiba, the State capital (**Figure 1**), in Southern Brazil. Since its foundation in 1912, the city’s economy has passed from being agricultural to industrial. It occupies an area of 1,343 km^2^ and has a population of 41,257 inhabitants; population density is 30.75 inhabitants/km^2^. São Mateus do Sul is characterized by a subtropical climate given its altitude of 835 m a.s.l. Apart from the two most important industrial plants (shale oil and ceramic), 100 small industries account for 60% of the local economy, while agriculture and services represent 40% (Instituto Brasileiro de Geografia e Estatística [IBGE], 2010).

### Tree Bark and Shale Sampling

**FIGURE 2:**
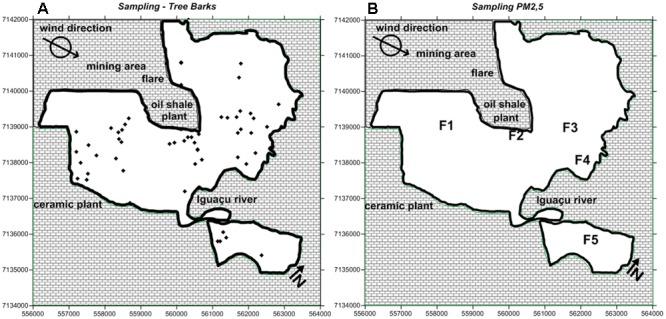
**(A)** Distribution of 60 sampling sites for tree bark collection; **(B)** representation of wind direction and the five georeferenced sites (F1–F5) for PM_2.5_ sampling.

Approximately 60 sampling sites were selected across the city of São Mateus do Sul (**Figure 2**). For each sampling site, the precise coordinates were recorded using a Global Position System for the Universal Transverse Mercator (UTM) coordinate system. The strategy for collecting was based on the main wind direction (W-E) around the shale plant. Rough tree barks from *Araucaria angustifolia* (commonly known as Parana-pine or Brazilian-pine) were taken for the chemical analysis. This is the predominant tree species in São Mateus do Sul (Barbieri and Heiden, 2012).

Diameter at Breast Height (DBH) method was used to ensure homogeneity of the sampling. Since bark contamination from soil inputs is limited to 1.5 m the length of the trunk, it has been assumed that above this level, air becomes the main source of pollutants in bark (Wolterbeek and Bode, 1995; Schelle et al., 2008). For this reason, a special attention was given in order to all the bark samples were taken from trees with average perimeter of 2.0 and 1.5 m above ground, instead of 1.30 m, that is commonly used for DBH (Clark et al., 2001).

Further, some rough bark trees from Caucaia do Alto city, located in a rural area 50 km from São Paulo city, have been analyzed given that the region is considered a control area for evaluating the levels of the potential pollutants under sudy. According to Guimarães et al. (2000), the level air cotaminants does not represent a health risk for the inhabitants of Caucaia do Alto.

Some samples of retorted shale (*n* = 5), a solid residue obtained from the thermal transformation of oil shale, were taken from the area surrounding the refinery to be analyzed and compared with the elemental data of the tree bark.

### Particulate Matter Sampling

Fine particulate matter was sampled and collected from five georeferenced sampling sites with an aerodynamic diameter below 2.5 μm (PM_2.5_); wind direction (W-E) was also considered (**Figure 2**). The PM_2.5_ samples were collected on 0.8-μm and 37-mm polycarbonate filters (Isopore^TM^ Membrane Filters Polycarbonate, Millipore, United States) using Harvard Impactors (Air Diagnostics, Harrison, ME, United States) operating at 10 L min^-1^ (Mauad et al., 2008) over a 24-h sampling period of 5 days. The PM_2.5_ facilities used in São Mateus do Sul belong to the Faculty of Medicine, University of São Paulo.

Although PM_2.5_ measuring is an expensive method, its application was also necessary to validate the geostatistical approach (Wasserman and Queiroz, 2004; Ribeiro et al., 2013) and enabling its utilization in future low-cost air pollution studies (Brown et al., 2007). Accordingly, given that PM_2.5_ measuring makes use of facilities and staff to ensure the safety of the equipment and reliability of the data generated, which make it a costly procedure as mentioned earlier, the financial resources and trained staff are not yet available in the study area. As a result, the PM_2.5_ measuring was carried out by using only the five sample sites in São Mateus do Sul, emphasizing the regions closer to the shale refinery and ceramic plant and others more distant from the industrial areas.

### Preparation and EDXRF Analysis

Tree bark samples were removed using a sharp knife and stored in sealed brown paper envelopes. The green layer of lichens and mosses was removed from some of the bark samples, and the outer layer of the bark (∼3 mm thick) was analyzed (Schelle et al., 2008). The samples were not washed for the purpose of measuring the elements that were physically trapped on the surface of the bark. Both tree bark and retorted shale samples were cleaned using a soft nylon toothbrush. Afterward, they were grated using a titanium grater, ground and sieved to obtain small-sized grains (maximum 0.2 mm). About 0.5 g of each sample was weighed for the analysis. The powdered material was pressed (4 t/load) with approximately 2.5 g of boric acid (reagent with analytical purity grade) to produce a pellet that was analyzed using an Energy Dispersive X-ray Fluorescence Spectrometer (EDXRF-720/800HS, Shimadzu Corporation, Japan). The measurement parameters were: time (180 s), target (Rh, 50 kV × 100 μA) and Si (Li) detector. The calibration curves were adjusted by linear regression using specific parameters of the equipment to correct the matrix effects. EDXRF was employed to determine the composition of the tree bark, retorted shale and particulate matter (PM_2.5_), according to Richardson et al. (1995).

### Attenuation of the Concentration Model

The USEPA defines natural attenuation as “the naturally occurring processes in soil and groundwater environments that act without human intervention to reduce the mass, toxicity, mobility, volume, or concentration of contaminants in those media” (United States Environmental Protection Agency [USEPA], 1987).

Although this phenomenon is preferred over human intervention to restore polluted areas, it rarely happens. Therefore, several approaches have been conceived to assess environmental impacts caused by anthropogenic activity (Nowak and Crane, 2000; Fenn et al., 2009).

In this work, we used the attenuation of the concentration model of the element based on the studies by Wasserman and Queiroz (2004) and Ribeiro et al. (2013). The model evaluates the spatial distribution of elements on the natural surface and generates values that describe the reduction in levels of elements from a “hot spot” (point of high concentration) in different directions, thereby simulating the destination of movement of the element in the study area. Accordingly, from the pollutant’s concentration data it is possible to assess the element’s behavior in a particular ecosystem and estimate pollutant mobility. Attenuation (A) values are given according to the following equation (Ribeiro et al., 2013):

$$\begin{array}{c} A=gradF=\left(\frac{\partial E}{\partial x}\right)+\left(\frac{\partial E}{\partial y}\right) \end{array}$$

where

A = attenuation of concentration in (μg/g)/m

E = concentration of an element E in (μg/g)/m

grad F = concentration gradient of an element E in (μg/g)/m


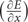
 = longitudinal derivative of the concentration of an element E in (μg/g)/m


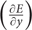
 = latitudinal derivative of the concentration of an element E in (μg/g)/m.

From the A values, it is possible to elaborate a map that indicates the spatial behavior of an element and its range from the hot spot(s). This, in turn, indicates the element’s mobility and retention areas (higher values of A), which may be associated with singular characteristics (chemical, geological or physical variables) at the investigated area and highlight the main source of the element (Ribeiro et al., 2013).

By using this model in the study area, for the elements investigated, it was expected that the maps could more effectively exhibit the main source of air contaminants and the local sites characterized by their marked retention in conformity with PM_2.5_ maps, World Health Organization [WHO]se information has commonly been accepted in studies evaluating air pollution.

Surfer^®^ 8.0 (GoldenSoftware) was used to create all the maps in this study. The contour lines were interpolated by the simple Kriging method (Matheron, 1971). Excel software (Microsoft, version 2010) was used in the attenuation of the concentration model as well.

### Statistical Analyses

To compare the concentrations of elements between tree bark samples from São Mateus do Sul and samples from Caucaia do Alto, the statistical *t*-test was applied using the STATISTICA^®^ 8.0 software for Windows. To identify the possible source of pollution, the multivariate statistical analysis – factor analysis (FA) with Extraction with Principal Components – was applied (Yeomans and Golder, 1982; Johnson and Wichern, 1992), using STATISTICA^®^ 8.0 for Windows.

The possible association between gradients of pollutants in tree bark and the frequency of respiratory diseases was evaluated using one-way ANOVA across four categories (quartiles) of element accumulation in the barks. ANOVA was followed by Tukey’s and Bonferroni’s *post hoc* tests (Calado and Montgomery, 2003).

### Health Outcomes

The health records were obtained under supervision of a nurse assistant, World Health Organization [WHO] belonged to the technical staff of the Basic Health Unit of São Mateus do Sul. The ratio between respiratory/non-respiratory (disease) was calculated for the period between 1997 and 2006. Of the 3000 records, only 245 patients had provided their residential address, which was a key parameter to plot the maps. By using a tool available in the SURFER program, the geographical coordinates (UTM) for each patient address were identified on the city map.

This database is public and can also be obtained from the Information Technology Department of the Public Health Care System -SUS (DATASUS), Brazilian Ministry of Health (Brasil - Ministério da Saúde, 2007). According to the Brazilian laws, researches based on public information, without possibility of individual identification, can be developed with no approval of the National Committee for Research Ethics (Brasil, 2011, 2016).

## Results and Discussion

### Quality Assurance

The accuracy and precision of the tree bark analyses were checked by analyzing the elements that were found in retorted shale, which is the main solid waste surrounding a shale refinery. These elements are also found in standard reference materials (SRM): NIST 1547 Peach Leaves (**Table 1**), provided by the National Institute of Standards and Technology, USA, and Basalt Geological reference material - JB2 (**Table 1**) from the Geological Survey of Japan.

**Table 1:** Concentrations of elements obtained from NIST 1547 Peach Leaves, NIST 2783 Air Particulate Matter on Filter Media and from Basalt Geological JB2 and standard reference materials (SRM).

SRM	Element	Mean ± SD^a^	RSD^b^ (%)	RE^c^ (%)	Values of certificate
Peach Leaves NIST 1547 (μg g^-1^)	Cd	n.d^d^			0.026 ± 0.003
	Cr	1.00 ± 0.06	6.0		1^e^
	Cu	3.7 ± 0.2	5.4	1.0	3.7 ± 0.4
	Fe	219.7 ± 14.0	6.4	0.8	218 ± 14
	Mn	97.9 ± 10.6	10.8	0.1	98 ± 3
	Ni	n.d^d^			0.69 ± 0.09
	Pb	0.84 ± 0.16	23.8	3.4	0.87 ± 0.03
	S (%)	0.20 ± 0.08			0.2^e^
	V	n.d^d^			0.37 ± 0.03
	Zn	18.0 ± 1.0	5.6	0.6	17.9 ± 4
Air Particulate Matter NIST 2783 (ng cm^-3^)	Fe	27500 ± 4800	17.4	3.7	26500 ± 1600
	S	1050 ± 30	2.8	2.1	1050 ± 260
	Si	58500 ± 315	0.5	0.2	58600 ± 1600
Basalt JB2 (%)	SiO_2_	53.3 ± 0.4	0.7	1.5	52.54 ± 0.03

*^a^Arithmetic mean and standard deviation; ^b^relative standard deviation; ^c^relative error, ^d^not determined; ^e^information value.*

The accuracy with relative errors for Cu, Fe, Mn, Pb, and Si was lower than 3.4% while the precision with relative standard deviations (RSD) was lower than 6.4%, except for Mn and Pb with RSD around 11 and 24%, respectively. The accuracy for S and Cr was not calculated, since these elements present only information values in the SRM. Considering that only the levels of Fe, S, and Si in the retorted shale samples were significantly higher than those found in tree barks from Caucaia do Alto, the precision and accuracy of the results for the particulate matter were verified solely for these elements. The results for SRM-NIST 2783 Air Particulate Matter on Filter Media were considered satisfactory and are presented in **Table 1**. The relative errors for Fe, S, and Si were lower than 3.7%, and RSD were lower than 2.8, except for Fe with an RSD around 17.4%.

### Retorted Shale, Tree Bark and PM_2.5_ Results

The main chemical elements found in the retorted shale samples were Cr, Cu, Fe, Mn, Pb, S, Si, and Zn; their average concentrations (μg g^-1^) and standard deviations are shown in **Table 2**.

**Table 2:** Concentrations of elements in the retorted shale samples.

Elements (μg g^-1^)	Mean ± SD
Cr	21 ± 1
Cu	32 ± 1
Fe	21445 ± 15
Mn	130.03 ± 0.02
Pb	15.1 ± 0.1
S	11335 ± 10
Si	118422 ± 54
Zn	28 ± 1

**Table 3** reports the concentrations of the elements (i.e., range, average, median and standard deviation) for about 60 tree bark samples from São Mateus do Sul and Caucaia do Alto.

**Table 3:** Concentrations of elements in tree bark from São Mateus do Sul and Caucaia do Alto.

Elements μg g^-1^)	Sampling Site					
	São Mateus do Sul, PR^∗^			Caucaia do Alto, SP^∗∗^		
	Mean ± SD	Median	Min-Max	Mean ± SD	Median	Min-Max
Cr	17 ± 12	14	7–83	17 ± 14	12	6–49
Cu	32 ± 10	31	16–60	31 ± 10	27	21–46
Fe	4177 ± 3175	2909	525–16528	888 ± 337	704	618–1553
Mn	335 ± 176	287	135–839	294 ± 160	219	146–548
Pb	13 ± 7	11	3–48	10 ± 4	9	6–15
S	2429 ± 664	2382	1469–3760	1202 ± 64	1210	1077–1270
Si	14890 ± 2698	11012	558–71738	722 ± 450	632	174–1168
Zn	29 ± 14	26	3–86	23 ± 14	19	9–48

*^∗^PR, Paraná State; ^∗∗^SP, São Paulo State.*

**FIGURE 3:**
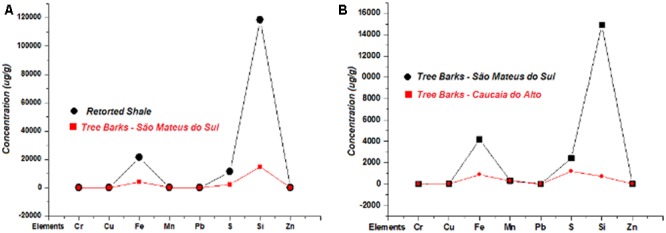
**(A)** Comparison of element concentrations found in tree bark in the study area and in retorted shale; **(B)** comparison of element concentrations found in tree bark in the study area and in Caucaia do Alto, São Paulo State.

By comparing the concentrations of the elements found in the retorted shale with those in tree bark from São Mateus do Sul, it is possible to observe that only the Fe, S, and Si levels in retorted shale were higher than their levels in the tree bark samples (**Figure 3**). Also, the Fe, S, and Si levels in the tree bark samples from the same study area were much higher than their levels in the tree bark samples from Caucaia do Alto, which is considered the control region for this biomonitoring study. Student’s *t*-test (with a significance level ≤ 0.05) indicated that the concentrations of these elements are statistically different. In contrast, the Cr, Cu, Mn, Pb, and Zn concentration levels in the study site are in agreement with the levels determined in the samples from the control region (**Figure 3**); i.e., their results were not relevant to suggest an anthropogenic impact in São Mateus do Sul, but they will be used for the multivariate statistical approach. Therefore, based on the results it seems that the shale refinery is the main source of Fe, S, and Si contents in São Mateus do Sul.

A comparison of the concentration levels, for Fe and S, obtained in this study with those of similar studies in other cities is reported in **Table 4**. The concentrations for both elements are much higher in São Mateus do Sul than in large cities around the world. For instance, Schulz et al. (1999) conducted a temporal (1987 and 1996) study in East Germany on Scots pine bark to monitor several pollutants, including Fe. The Fe contents were approximately five times lower (3490 μg g^-1^) than some Fe levels (16528 μg g^-1^) observed for the barks collected surrounding the shale refinery. Schulz et al. (1999) analyzed tree bark collected in the industrial regions around the cities of Leipzig, Halle, and Bitterfeld, which (unlike São Mateus do Sul) comprise several huge industrial facilities of the steel, metallurgical and chemical sectors.

**Table 4:** Literature data for Fe and S concentrations (μg g^-1^) in tree bark.

Other countries (large cities)	Fe (Mean ± SD or Range)	S (Mean ± SD or range)
Czech Republic (Böhm et al., 1998)	2917	1035
Northern Finland and the Kola Peninsula (Poikolainen, 1997)	102 ± 67	373 ± 71
Germany (Schulz et al., 1999)	3490 ± 210	#
United Kingdom (Schelle et al., 2002)	147 – 3570	#
Argentina (Fujiwara et al., 2011)	454.5 – 1230	#
Bosnia and Herzegovina (Škrbiæ et al., 2012)	184 – 1648	#

*^#^Data not available.*

The comparison of our study results with the levels of pollutants with PM_2.5_ results from other studies also emphasizes how allarming the issue of air pollution is in São Mateus do Sul. **Table 5** presents the analytical results for PM_2.5_ from the five sampling sites around the city. The levels of Fe and Si are in the same order of magnitude as those found in Barcelona, Mexico, and Seoul (Querol et al., 2001; Chow et al., 2002; Kang et al., 2004). The *S* values are slightly lower than those in Seoul (Kang et al., 2004), which is one of the most densely urbanized areas in the world with 52500 inhabitants per square mile (Jun et al., 2013). Therefore, these element concentrations point out that although São Mateus do Sul is a small city, where agricultural activities are also economically relevant, it has expanded rapidly with inhabitants settling around the refinery facilities. As a result of this unplanned urbanization, the entire city has been suffering from the same adverse effects that can be observed in megacities around the world.

**Table 5:** Concentrations of Fe, S, and Si in PM_2.5_ samples.

Local	Fe (ng m^-3^)	S (ng m^-3^)	Si (ng m^-3^)
Site F1 in SMS	327	430	798
Site F2 in SMS	549	900	718
Site F3 in SMS	111	733	171
Site F4 in SMS Site F5 in SMS	136 104	568 397	247 329
Los Angeles (Chow et al., 1994)	99	#	52
Ch’ongyu (Kang et al., 1997)	146	#	360
Barcelona (Querol et al., 2001)	260	#	490
México City (Chow et al., 2002)	560	#	#
Seoul (Kang et al., 2004)	555	3163	1361

*SMS São Mateus do Sul - this study; ^#^data not available.*

### Distribution Maps of Fe, S, and Si Concentrations in Tree Bark and PM_2.5_

**FIGURE 4:**
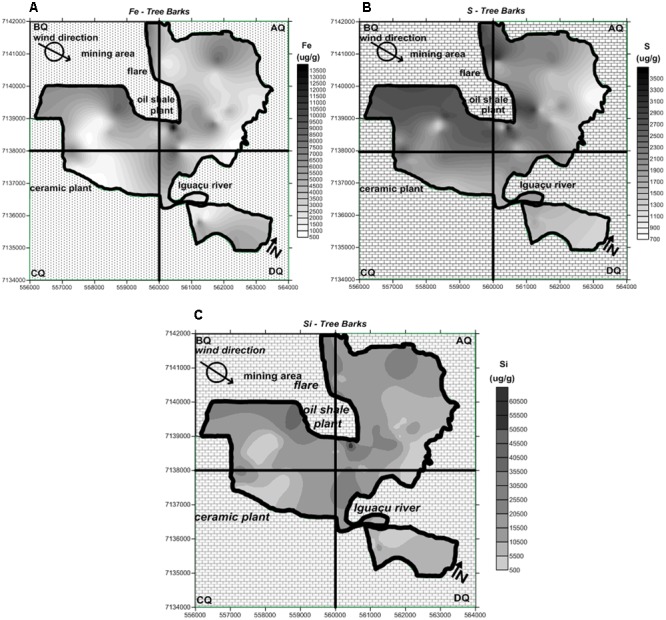
Distribution map of the **(A)** Fe, **(B)** S, and **(C)** Si concentrations found in tree bark in the study area.

The map of São Mateus do Sul was divided into quadrants labeled AQ, BQ, CQ and DQ to view the city in regions. As shown in **Figure 4A**, Fe concentrations vary across the city and tend to exhibit higher concentrations at the downwind borders of the shale plant. The chemical composition of shale particles consists partly of Fe (Costa-Neto, 1983; Pimentel et al., 2006).

**Figure 4B** shows the distribution of S concentrations in tree bark collected in São Mateus do Sul. The influence of emissions from the shale oil company seems to be more evident when S is considered as its tracer. Likewise Fe, S, and Si are also chemical constituents of shale residues (Costa-Neto, 1983; Pimentel et al., 2006).

**Figure 4C** shows the distribution of Si concentrations in tree bark collected in the study area. The area of influence of Si is located downwind of the mining area, with a more restricted spatial distribution than that of S and Fe, probably reflecting the higher granulometry of particles generated during the drilling process or the reduced height of the emission source (ground level at the mine vs. chimney in the case of S).

**FIGURE 5:**
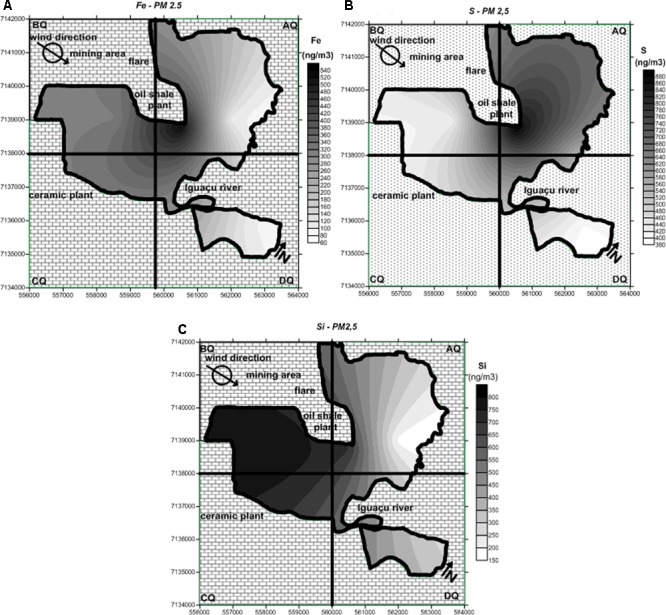
Distribution map of the **(A)** Fe, **(B)** S, and **(C)** Si concentrations found in in PM_2.5_ in the study area.

The PM_2.5_ maps for the Fe, S and Si concentrations are illustrated in **Figure 5**. Because of the weakness in the number of sampling sites, the spatial distribution extrapolated by the Kriging method may lead to uncertain findings, which should be analyzed to evaluate the application of the attenuation of the concentration model. In general, all portions of the map seem to have been affected by the air contaminants. Even so, the highest Fe contents were observed mainly at the map quadrants AQ and BQ, and slightly at CQ (**Figure 5A**). The AQ quadrant seems to be more affected by S derived from the atmosphere (**Figure 5B**). The assessment of Si contamination reveals that its highest values were found at BQ and CQ. The dataset obtained from the PM_2.5_ analysis indicates that the regions in the vicinities of the two largest industries (shale refinery and ceramic plant) of São Mateus do Sul (**Figure 5**) were more affected by anthropogenic activity.

### Maps of Attenuation of Fe, S, and Si Concentrations in Tree Bark Samples

**FIGURE 6:**
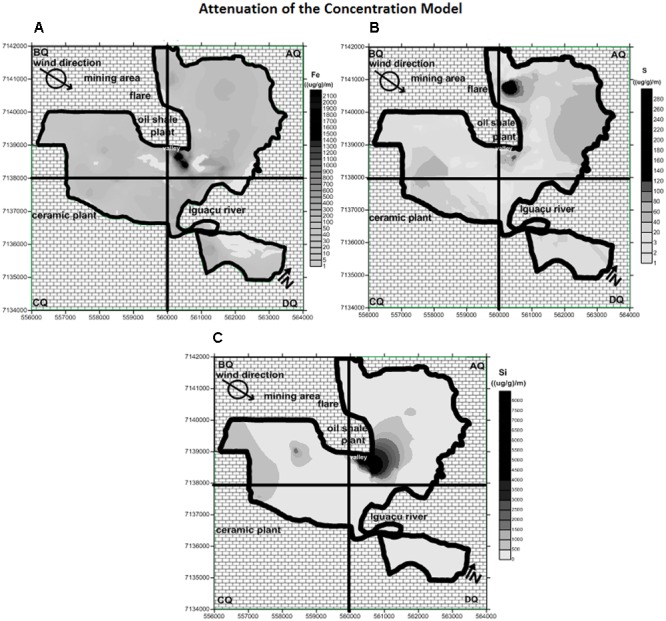
Maps of the attenuation model showing hot spots for the **(A)** Fe, **(B)** S, and **(C)** Si concentrations in the study area.

From the results of the Fe, S, and Si concentrations in tree bark samples, it was possible to apply the attenuation of the concentration model. The attenuation maps obtained for Fe, S, and Si, respectively, are shown in **Figure 6**. The maps of Fe concentration in tree bark (**Figure 4A**) and in PM_2.5_ (**Figure 5A**) provided a general impression of the high concentrations of this metal around the refinery facilities.

By applying the geostatiscal model, it is possible to observe the point where Fe was strongly retained (**Figure 6A**). The map showed that the hot spot of Fe attenuation was found close to the main source (shale refinery), in accordance with the highest Fe concentration shown in **Figures 4A, 5A**. Data from the attenuation maps indicate that Fe had low mobility precisely in a lower region in São Mateus do Sul, suggesting that low altitude acts as a geological barrier for the metal.

The attenuation map of S emphasized two hot spots in the AQ quadrant (**Figure 6B**): a slight hot spot, also located in the valley region, and an intense hot spot found in the northern portion. In this region the flare device is located, which functions as a continuous source of S emissions into the environment. During the refining process, shale is heated at high temperatures producing oil, gas, and sulfur. Therefore, although the movement of air is intense, it is not enough to dissipate the sulfur. As a result, the element is deposited close to the shale plant.

The highest values from the PM_2.5_ measurements (**Table 5**) corroborate the data obtained from the attenuation of concentration model. Furthermore, despite their weakness, PM_2.5_ maps could provide an overview of the highest concentrations of S (**Figure 5B**), World Health Organization [WHO]se regions include the highest attenuation values (hot spots).

For Si, the attenuation map indicates a hot spot in the same valley of the city (**Figure 6C**), but only with a larger coverage than Fe retention. The prevailing wind direction in the region is West to East (W-E), i.e., Si emissions from the ceramic industry and the mining process should be carried to the eastern part of the city. However, due to the topographic depression (natural barrier), the air remains trapped. The attenuation map of Si is consistent with the information obtained from the distribution of Si concentrations in the PM_2.5_ map (**Figure 5C**). Because geological features prevent the diffusion of the contaminants, neighboring dwellers may be more affected by the air pollution.

### Multivariate Statistical Analysis

Multivariate statistical analysis, Factorial Analysis (FA) with extraction by principal components, is a powerful pattern recognition technique that attempts to explain the variance of a dataset of inter-correlated variables with a smaller set of independent variables (Yeomans and Golder, 1982; Johnson and Wichern, 1992). Mathematically, FA searches for such joint variations in response to unobserved latent variables through the description of the measured variables according to the linear combination of potential factors, plus error terms. Therefore, FA indirectly determines the association between elements that correspond to the main elements observed and generated by diverse emissions sources (Johnson and Wichern, 1992).

Twelve elements formed two groups of factor correlations (**Table 6**). According to the Kaiser criterion (Yeomans and Golder, 1982) used to assess the results, two main components were considered which accounted for 71% of the total variance (**Table 6**).

**Table 6:** Factor loadings eigenvalues and total variance.

Variables	F1	F2
Cr	0.862575	#
Cu	0.599942	#
Fe	#	0.757039
Mn	#	0.620149
Pb	0.902466	#
S	#	0.828164
Si	#	0.895939
Zn	0.927540	#
Eigenvalues	3.901023	1.785852
Total variance (%)	48.76279	22.32315

*^#^Data not available.*

The matrix of the components for the dataset indicated that Cr, Cu, Pb, and Zn presented good correlations and were associated with the first component (F1), with a total variance of 49%. Fe, Mn, S, and Si were grouped with the second factor (F2), with a total variance of 22%. F1 grouped the metals that in the pattern of soil geochemistry are termed trace elements and are also commonly related to anthropogenic activities, such as vehicular emissions (Oliveira et al., 2002; Wang et al., 2003; Assunção, 2004; Ravindra et al., 2004; Bergamini et al., 2006). Nevertheless, the Cr, Cu, Pb, and Zn concentrations in the bark samples from São Mateus do Sul were similar with those found in the bark samples from Caucaia do Alto (**Table 3** and **Figure 3**), suggesting that these metals are associated with natural sources in the study region. F2 grouped the elements considered, together with others, as major components of the soil matrix (Osman, 2012). In reality, the soil matrix consists of soil fractions which include organic soil, inorganic non-crystalline material and inorganic crystalline material. These latter two include components like the oxides and hydrous-oxides Fe, Al, Mn, and Si, primary and clay minerals, carbonates, sulfates, phosphates and sulfides (Yong and Mulligan, 2003). Thus, the Fe, Mn, S, and Si found in particulate matter deposited on tree bark could also be associated with natural sources. However, except for Mn, the other elements presented the highest concentrations in the shale and tree bark samples and exceeded their levels in the samples from Caucaia do Alto (**Table 3** and **Figure 3**). Moreover, the Fe, S, and Si levels in PM_2.5_ were in conformity with their levels in polluted areas (**Table 5**). According to Ying-Mei et al. (2009), in regions where there is industrial mining the main source of Fe, S, and Si may be associated with burning ore. Ots and Reisner (2007) also reported that S is produced during the burning of tailings in mining industry. Therefore, although statistical analysis grouped the major elements of the soil matrix in F2, it seems that the main sources of Fe, S, and Si are from the two most important industries (ceramic and shale mining) of São Mateus do Sul.

### Health Outcomes

The size of the PM is the main factor responsible to the intensity of the health problem, although the exposure effectiveness to PM also depends on the physical characteristics of each individual (Brown et al., 2013). The most impact on human health are associated to PM with an aerodynamic diameter below 10 μm, because of their capapability to penetrate deeply into respiratory tract, causing a greater inflammatory response (Nemmar et al., 2001). For PM_2.5_, they are able to reach the respiratory bronchioles and the alveoli where gas exchange occurs (Löndahl et al., 2006).

**FIGURE 7:**
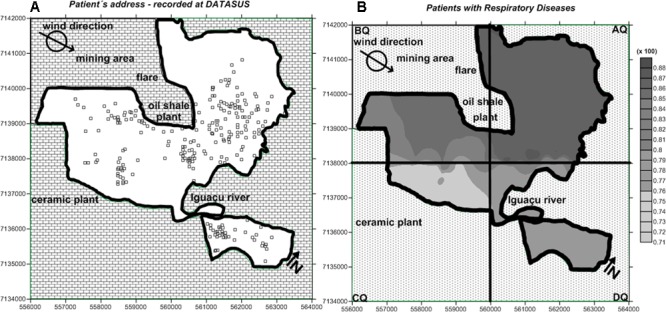
**(A)** Residential location of the 245 patients selected in the present study; **(B)** distribution map of respiratory diseases.

**Figure 7A** shows the residential sites of the 245 patients in the study. For each quadrant, the relative number of patients with respiratory disease was calculated (**Table 7**).

**Table 7:** Number of patients with respiratory disease in regions per quadrants (Q) of the study area.

Quadrant	Total number of patients per quadrant	Absolute number of patients with respiratory disease	Relative number of patients with respiratory disease (%)
AQ	115	100	87
BQ	48	41	85
CQ	23	17	74
DQ	59	46	78

Bioassay on redox activity in particulate matter toxicology indicated that the production of reactive oxygen species (ROS) was significantly positively correlated with the contributions of three main sources: Fe, soil dust and water-soluble carbon. ROS is constantly produced in the human body as the natural consequence of aerobic metabolism, and is integral for maintaining tissue oxygen homeostasis (Zhang et al., 2008). An overproduction of ROS caused by Fe was responsible to promote some kind of damage to living cells and tissues, which was enough to initiate an inflammatory process in living organism (Kadiiska et al., 1997).

In the case of air pollution in São Mateus do Sul, the regions with the highest prevalence of respiratory diseases are located in quadrants AQ and BQ (**Figure 7B**). Nonetheless, the number of cases observed in the CQ and DQ quadrants was slightly lower, suggesting that the population of the entire city was experiencing respiratory distress.

A study was performed in Alvarez City (located in the dry area of Iran) to evaluate the number of hospital admission for respiratory disease (HARD) from human exposure to sulfur dioxide. The authors also observed that the number of hospitalization was maximum among the citizens World Health Organization [WHO] lived close to heavy industry (steel, oil, and gas) areas with high sulfur dioxide emitters. An increase of 10 μg m^-3^ in sulfur dioxide level led to an increase of 3.4% in the HARD (Goudarzi et al., 2016).

Although the complexity of epidemiological studies focused on air pollution, there is no doubt that chemical contaminants are carried in the atmosphere as gases, aerosols, and particulate matter and may be transported over large distances causing local, regional or global impacts, even in pristine areas such as the polar regions (Boutron et al., 1994; Kim et al., 2015). Even so, the map generated using the health data was consistent with the information obtained from the Fe, S, and Si distribution maps for tree bark, the PM_2.5_ samples and attenuation of the concentration modelattenuation of the concentration model (**Figures 4–6**, respectively).

**FIGURE 8:**
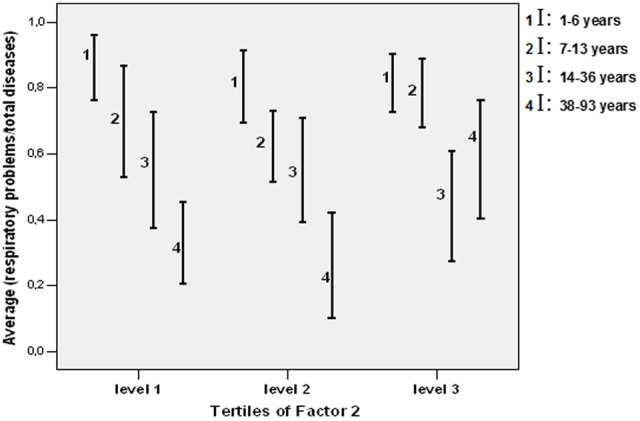
Ratio values of respiratory disease/total diseases (standard error) according to the categories of Factor 2 (Fe, S, and Si) disaggregated by quartiles of age-groups.

By applying ANOVA, the coefficients of Factors 1 and 2, obtained during FA for each sampling site, were categorized in levels (tertiles). The frequency of respiratory disease (respiratory/total) was considered the dependent variable of tertiles of each factor and the four age categories (1–6, 7–13, 14–36, and 38–93 years of age). F1 did not exhibit any significant statistical association with health outcomes. On the other hand, the frequency of patients older than 38 years increased with increasing contributions of F2 at their residency site (*p* < 0.05). The correlation between F2 and age is shown in **Figure 8**.

The combination of spatial characterization of pollution, clinical data and the inferential statistics approach revealed adverse effects only in the older population. This finding may be the result of two non-exclusive events. One possibility is that respiratory events are quite common in children, and thus the influence of air pollution is masked by the “high noise” in the signal. On the other hand, it may be suggested that it takes shale oil emissions longer to induce adverse effects, as the result of cumulative exposure and decreased respiratory defense with age.

The exposure to PM_2.5_ is statistically significant to cause cardiovascular and respiratory hospitalizations, reducing the life expectancy of the population by about 8.6 months on average. The elderly people are more susceptible to these illness (Dominici et al., 2006; Krewski, 2009).

Moreover, it sould bear in mind that the economic impacts related to airborn epidemic diseases are also significant. For the year 2009, the economic loss related to the Chinese exposure to air pollution was estimated in US$ 106.5 billion (Hou et al., 2012).

From these perspectives, a possible alternative to remedy the problem would be the introduction of green infrastructure, which could filter out some atmospheric contaminants (Sanesi et al., 2016). The role of vegetation cover in reducing PM_2,5_ concentrations in Nanjing, China was also discussed by Chen et al. (2016).

Accordingly, for the scenario observed in São Mateus do Sul it is plausible to consider older individuals as the better bioindicators for the adverse effects caused by shale industry emissions. However, the health outcomes of São Mateus do Sul are limited, since epidemiological studies must take into account pre-existing illnesses, human exposure to contaminants from air, soil, drinking-water and food, and in-depth knowledge about the principle of causality (Kundi, 2006), which could not be considered in this study.

## Conclusion

The bioindication method (using tree barks) to assess the air quality can be considered a comprehensive, precise, inexpensive, and easy strategy to handle and implement. It is important that developed alternatives to estimate the level of contamination do not depend on sophisticated instrumental methods that require substantial resources for their purchase and, above all, long-term maintenance.

Further, the results from the spatial distribution in PM_2.5_ enabled to validate the concentration attenuation model, which highlighted the hot spot retention of Fe, S, and Si, also indicating the presence of a valley region which acts as an orographic barrier and obstructs the spread of these air pollutants throughout the city.

According to Hopke (2009), the atmosphere is a very complex system and the air quality managements are usually based on computer models which take into account information on the levels of local potential contaminants. Alternatively, the majority of air quality models uses chemical composition in particulate matter, which indicates the main polluting source surrounding the investigated area. Therefore, the attenuation of the concentration model is in agreement with this premise.

Further, the findings from the attenuation of the concentration model may explain why the cases of respiratory disease were observed mainly in those patients World Health Organization [WHO] live in areas contiguous to the geochemical barrier. Therefore, the protocol designed to monitor the air quality of São Mateus do Sul using the combined biomonitoring/geostatistical approach and health data highlighted the main sources of atmospheric contaminants in the city and neighboring regions. For this reason, it may be useful to conduct environmental screening in areas with aerial emissions (pollutants as oxides, dioxides, particulate matters and others) and lacking the conventional networks for air pollution monitoring. Moreover, Lafortezza et al. (2013) stress the importance of urban green infrastructure, which may supply ecosystem services and promote human health and wellbeing. In this sense, trees in the urban enviroment might be used not only as bioindicators of air pollution but also as important tools for filtering pollution and cooling.

The rapid and unplanned growth observed in São Mateus do Sul could be characterized as a process of land development. This would imply the causes and consequences of particular land-use behaviors, which in the future may lead to urban sprawl as pointed out by Galster et al. (2001).

However, since the early 2000s, the population of São Mateus do Sul has taken a firm stand to ensure that the city’s growth is guided by sustainable planning and management. Because of the benefits generated by the industrial sector, its activities normally affect urban planning policies creating a conflict of interests and divergent moral and political viewpoints. Thus, in the last 10 years the authorities have made a special effort to form partnerships with different stakeholders (e.g., public administration/university) to ensure that industries meet their social and environmental responsibilities for securing quality of life for the city’s inhabitants.

This concern of local residents with their well being has caused an improvement to the São Mateus Master Plan, which might lead to sustainable development for the city. Furthermore, based on the information from our study, the Paraná authorities could request the shale refinery to bear the costs of a more detailed environmental study focusing on the toxicity of pollutants and human exposure and indicating remediation measures to minimize the effects of emissions produced by human activity surrounding industrial sites and bordering São Mateus do Sul. In light of the above, and despite the lack of evidence on the adverse effects of mining activities on human health, the results of this air pollution study provide scientific support for sound decision making.

## Author Contributions

AF: design and planning of the research; sample collection, analysis and interpretation of the dataset. AR: analysis and interpretation of the dataset, elaboration of the draft, critical review of the content and construction of the final version of the manuscript. MF: analysis and interpretation of the dataset, elaboration of the draft, critical review of the content and construction of the final version of the manuscript. CK: critical review of the content and elaboration of the manuscript draft. CQ: analysis and interpretation of the dataset, elaboration of the draft, critical review of the content and construction of the final version of the manuscript. RL: analysis and interpretation of the dataset, elaboration of the draft, critical review of the content and construction of the final version of the manuscript. JS: analysis and interpretation of the dataset, elaborating of the draft, critical review of the content and construction of the final version of the manuscript. MS: design and planning of the research, analysis and interpretation of the dataset. PS: design and planning of the research, analysis and interpretation of the dataset.

## Conflict of Interest Statement

The authors declare that the research was conducted in the absence of any commercial or financial relationships that could be construed as a potential conflict of interest.
